# New Modified Recombinant Botulinum Neurotoxin Type F with Enhanced Potency

**DOI:** 10.3390/toxins13120834

**Published:** 2021-11-24

**Authors:** David Burgin, Cindy Périer, Gavin Hackett, Mark Elliott, Daniel Kwan, Fraser Hornby, Imran Mir, Jacquie Maignel, Sai Man Liu, Matthew Beard

**Affiliations:** 1 Ipsen Bioinnovation, 102 Park Drive, Abingdon OX14 3YS, UK; gavin.s.hackett@gmail.com (G.H.); mark.elliott@ipsen.com (M.E.); daniel.kwan@cytiva.com (D.K.); Fraser.hornby@ipsen.com (F.H.); i.mir992@googlemail.com (I.M.); sai.man.liu@ipsen.com (S.M.L.); Matthew.beard@ipsen.com (M.B.); 2 Ipsen Innovation, 5 Avenue du Canada, 91940 Les Ulis, France; Jacquie.maignel@ipsen.com

**Keywords:** botulinum neurotoxin, recombinant protein, potency, safety profile, in vivo

## Abstract

Botulinum neurotoxins (BoNTs) are notorious toxins and powerful agents and can be lethal, causing botulism, but they are also widely used as therapeutics, particularly to treat neuromuscular disorders. As of today, the commercial BoNT treatments available are from native A or B serotypes. Serotype F has shown efficacy in a clinical trial but has scarcely been used, most likely due to its medium duration of effect. Previously, the uniqueness of the light chain of the F7 subtype was identified and reported, showing an extended interaction with its substrates, VAMPs 1, 2 and 3, and a superior catalytic activity compared to other BoNT/F subtypes. In order to more extensively study the properties of this neurotoxin, we engineered a modified F7 chimera, mrBoNT/F7-1, in which all the regions of the neurotoxin were identical to BoNT/F7 except the activation loop, which was the activation loop from BoNT/F1. Use of the activation loop from BoNT/F1 allowed easier post-translational proteolytic activation of the recombinant protein without otherwise affecting its properties. mrBoNT/F7-1 was expressed, purified and then tested in a suite of in vitro and in vivo assays. mrBoNT/F7-1 was active and showed enhanced potency in comparison to both native and recombinant BoNT/F1. Additionally, the safety profile remained comparable to BoNT/F1 despite the increased potency. This new modified recombinant toxin F7 could be further exploited to develop unique therapeutics to address unmet medical needs.

## 1. Introduction

Botulinum neurotoxins (BoNTs) are highly potent neurotoxic proteins produced by some species of the genus Clostridia including *C. botulinum*, *C. butyrricum*, *C. baratii* and *C. argentinensis*. Conventionally, BoNTs are classified into seven different serotypes based on their serological typing and are designated as BoNTs A, B, C, D, E, F and G. These families can be further subdivided into sub-serotypes based on the specific amino acid variation. More recently, additional subtypes were identified [1,2], as well as a number of mosaic toxins [3] and some BoNT-like molecules, and even new BoNTs [4]. 

BoNTs are synthesised as large single chain precursors of approximately 150 kDa. The neurotoxin molecule is divided into two sub-units, an endopeptidase active light chain (LC, ~50 kDa) and a heavy chain (HC, ~100 kDa). The HC is made up of subdomains that confer the translocation and cell-specific binding activities. To reach the heterodimeric form, BoNTs undergo post-translational cleavage within a region called the activation loop, and the two resulting sub-units, LC and HC, remain associated through an interchain disulphide bond. The LC is a protease that cleaves N-ethyl-maleimide-sensitive factor attachment receptor proteins (SNARE proteins) inside targeted neurons. Each serotype targets specific SNARE proteins: serotypes: A and E cleave synaptosomal-associated protein 25 (SNAP-25) at different sites, C is capable of cleaving both SNAP-25 and Syntaxin, and BoNTs B, D, F and G cleave vesicle-associated membrane proteins 1, 2, and 3 (VAMP-1, -2, and -3). 

BoNTs have long been established as therapeutics and are able to treat a wide range of neuromuscular disorders and are used in multiple indications such as blepharospasm and spasticity [5]. These treatments utilise BoNT’s properties to inhibit the release of the neurotransmitter, acetylcholine, at the neuromuscular junction. Despite the variety of serotypes, as of today, A1 and B1 are the only ones used in clinical practice. Although they have a similar duration of action, B1 is used less as it requires an increased dose to achieve similar results. This is due to the low affinity of BoNT/B to the human receptor Syt II [5,6]. There are limited clinical trials involving other BoNT serotypes and recently, BoNTs A, B, C and F were investigated in healthy volunteers using extensive neurophysiological assessment [7,8]. Serotype E, which has a shorter duration of action compared to other serotypes, was also recently assessed in volunteers [9,10] as well as in neuromuscular pain conditions [11]. These studies confirm the preclinical results supporting the pharmacological differences between the serotypes. 

Though there is much conservation in the overall architecture and biology of the BoNTs, the different serotypes achieve their clinical effect by binding a range of cell surface receptors and cleaving different SNARE proteins. These differences confer to BoNT neuronal specificity and potency and lead to unique pharmacological properties such as the relative potency, duration of action and time to onset as reported in rodents pre-clinical studies [12,13]. Even relatively minor differences within subtypes can produce a change in these properties. For instance, BoNT/A3 was shown to have a significantly reduced duration of action in a functional mouse model compared to other BoNT/A subtypes [14].

BoNT/F is known to cause botulism in humans, with a few cases reported globally. At least two subtypes were isolated from botulism outbreaks including F1 and more recently F7. In a 2015 outbreak in France, *C. baratii* was isolated, identifying only one BoNT/F7 locus associated with orfX genes [15]. The BoNT/F serotype comprises a large number of sub-serotypes and shows greater sequence diversity than any of the other BoNT serotypes [16]. There are currently nine identified BoNT/F subtypes and experimental studies so far have shown marked differences in their enzymatic activity. Sikorra et al. reported on the differences in catalytic efficiencies, K_m_ and K_cat_ between the subtypes F1, F6, F7 and F9 [2]. Kalb et al. reported on their observation of the novel subtype F5 and the cleavage of a scissile bond between L54 and E55 of VAMP-2. The only other BoNT reported to cleave at this site is the mosaic BoNT/FA [17,18]. All other known BoNT/F subtypes cleave between Q58 and K59 of VAMP-2. BoNT/F7 has a more extensive interaction between its targeted substrate and exosites [19,20].

In order to more extensively study the properties of BoNT/F7, we engineered a recombinant version of BoNT/F7 (rBoNT/F7). During the engineering process, we subjected the toxin to post-translational activation with a variety of proteases. However, in all cases, we observed additional truncation outside of the activation loop. To overcome this, we created a chimeric protein mrBoNT/F7-1, which retains rBoNT/F7 activity but substitutes the rBoNT/F7 activation loop with that of BoNT/F1. In this study, we describe how modification provided a means to generate a highly pure heterodimeric toxin. We then took this into a suite of cell-free, in vitro, ex vivo and in vivo assays and demonstrated that the substitution resulted in an active and potent protein. Moreover, in these assays, the mrBoNT/F7-1 chimera showed an enhanced potency when compared to the native BoNT/F1 (nBoNT/F1) without altering its safety profile. 

During this study, we also explored the effect of the difference in ganglioside binding. Previously, Fu et al. showed that the binding domain of BoNT/F1 showed the following binding order: GT1b = GD1a ≫ GM3; no binding to GD1b and GM1a [21]. The group identified key residues required for binding within the ganglioside binding motif (GBM). However, it was later identified that additional residues outside of this motif were also important [22]. Scanning alanine mutagenesis and structural analysis showed Arg-1111 and Arg-1256 bound the Sia5 sugar and replacement with an alanine reduced binding by 8-fold and 22-fold, respectively. When aligning BoNT/F1 and BoNT/F7 Arg-1111 corresponds to Lys-1104, respectively (Figure S1). Though these residues are very similar there is a small difference with arginine offering greater stability to the protein. This small difference might produce a measurable effect in overall potency and thus further explain the differences in potency and other functional properties. To understand this possible effect, we created the mutant mrBoNTF1^R1111K^ to look for a change in potency. We show that this mutation does confer an increased potency in some of the assays used compared to both native and recombinant BoNT/F1.

## 2. Results

### 2.1. Expression, Purification and Proteolytic Activation of Recombinant BoNT/F7

**Figure 1:**
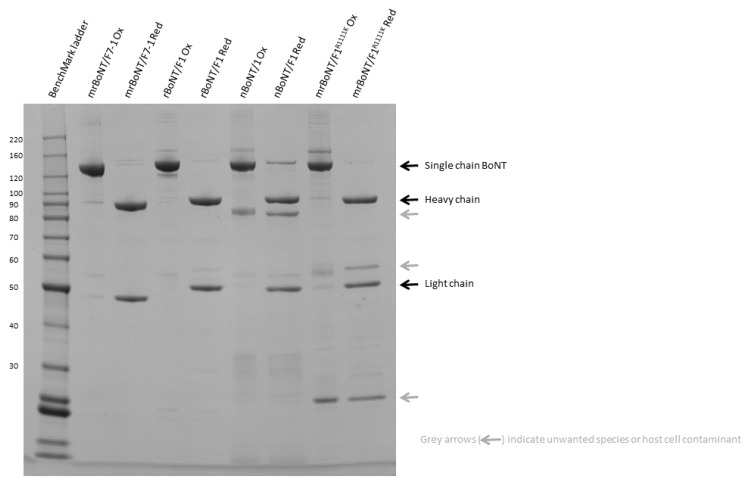
SDS-PAGE analysis of mrBoNT/F7-1 compared to nBoNT/F1 (metabiologics), rBoNT/F1 and rBoNT/F1^R1111K^. Samples with the suffix Ox are non-reduced and prepared using Tris-Glycine SDS sample buffer (Invitrogen), samples with the suffix Red were prepared in LDS sample buffer (Invitrogen) with the addition of 100 mM DTT.

The BoNT/F7 and BoNT/F1 gene sequences were codon-optimised for expression in *E. coli* and used to make five constructs: (1) rBoNT/F7 (prefix r indicates recombinant); (2) rBoNT/F1; (3) rBoNT/F1^R1111K^ which contains a point mutation in the ganglioside binding domain (R1111K); (4) mrBoNT/F7-1 in which the BoNT/F7 activation loop is replaced by that of BoNT/F1 (method previously reported by Hackett et al. [23]) (prefix m indicates modified); (5) mrBoNT/F7-1 (0) where (0) denotes endopeptidase inactive due to two point mutations in the light chain (E227Q and H230Y) (Figure 1).

rBoNT/F7(0), recombinantly expressed by *E. coli* and purified by hydrophobic and anion exchange chromatography, yielded between 2–3 mg/L of the single-chain form. However, we were unable to identify suitable conditions to generate the mature heterodimeric form of the protein. Several conditions and proteases were screened, three of which showed potential for further optimisation; these were Endoproteinase Lys-C (Lys-C), Endoproteinase Lys-N (U-Protein Express, Utrecht, The Netherlands) and recombinant trypsin (TrypZean^®^) tested between 0.63–20 µg/mL for cleavage of 0.25 mg/mL rBoNT/F7(0), 2 h reaction time at 37 °C. However, in each case, the protease generated unwanted cleavage products, also cutting at sites outside of the activation loop, assessed by SDS-PAGE, under all conditions tested (Figure S2). An alternative approach, to switch the activation loop to that of BoNT/F1 (UniParc sequence UPI00000B66D1) was therefore used. This approach was reported previously with work conducted on BoNT/FA [23]. This alternative construct mrBoNT/F7-1(0) behaved in a similar manner to rBoNT/F7(0) as the single-chain form during expression and purification and yielded final purified material with similar purity and profile on SDS-PAGE analysis (Figure 1). Conditions were successfully established for selective cleavage of the BoNT/F1 activation loop with Lys-C. Negligible truncated species were produced and a subsequent Phenyl HP chromatography step removed remaining impurities and/or truncates as well as residual Lys-C. The final activation conditions established were Lys-C at 0.1 µg/mL, mrBoNT/F7-1(0) at 0.33 mg/mL for 16 h at 4 °C. Confirmation of proteolytic cleavage in the activation loop was confirmed by N-terminal sequencing of the HC domain (Table 1) and intact mass spec of the reduced and non-reduced construct (Table 2).

This method was then used to purify and activate mrBoNT/F7-1 resulting in pure, well-activated material with a yield between 0.672–3.5 mg/L across three repeated batches. The methods for expressing, purifying and activating rBoNT/F1 and mrBoNT/F1^R1111K^ had been previously established in-house and are similar in approach to this expression, purification and activation for mrBoNT/F7-1.

### 2.2. VAMP-2 Cleavage

**Figure 2:**
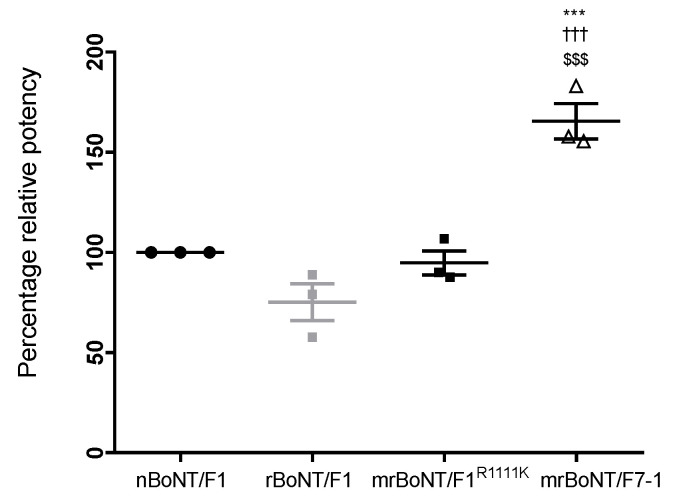
Comparison of potency in HPLC VAMP cleavage assay showing relative potency of rBoNT/F1, mrBoNT/F1^R1111K^ and mrBoNT/F7-1 normalised from nBoNT/F1 specific activity. *** *p* < 0.001 vs. nBoNT/F1, ^†††^ *p* < 0.001 vs. rBoNT/F1, ^$$$^ *p* < 0.001 vs. mrBoNT/F1^R1111K^.

The HPLC VAMP cleavage assay measures specific activity for cleavage of VAMP2-GFP by reverse phase HPLC; detecting the VAMP-2 substrate and cleaved product after incubation with active BoNT/F toxin. This allows the LC activity to be assessed in a cell-free environment. For each BoNT, the specific activity was determined and LC activity of rBoNT/F1, mrBoNT/F1^R11111K^ and rBoNT/F7-1 were expressed relative to nBoNT/F1 (Figure 2). 

rBoNT/F1 LC activity was found to be similar to nBoNT/F1. Likewise, mrBoNT/F1^R1111K^ LC activity appeared similar to both rBoNT/F1 and nBoNT/F1. Interestingly, mrBoNT/F7-1 LC activity was significantly higher than any of the BoNT/F tested (Table 3). 

No direct comparison can be made with other serotypes as this method was developed specifically to measure BoNT/F cleavage of VAMP-2. The BoTest Botulinum neurotoxin testing kit (Biosentinel, Madison, WI, USA), was tried initially. However, the reporter did not show any cleavage in the presence of rBoNT/F7. It is most probable that the substrate is too small to interact with as it has previously been reported by Kalb et al. [19]. The HPLC VAMP cleavage assay was thus developed and validated for BoNT/F sub-serotype activity.

### 2.3. Inhibition of Neurotransmitter Release in Rat Embryonic Spinal Cord Neurons

**Figure 3:**
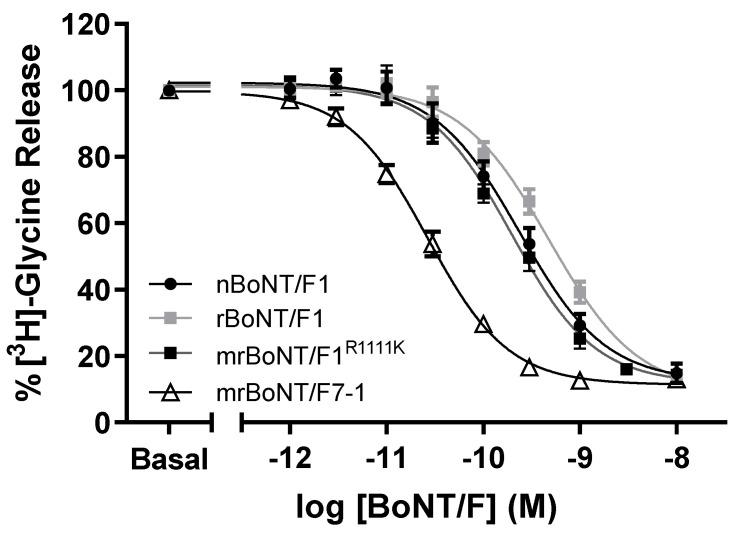
Inhibition of [^3^H]-glycine release from rat eSCN (**A**) Response curve following 24 h treatment with nBoNT/F1, rBoNT/F1, mrBoNT/F1^R1111K^ or mrBoNT/F7-1. Data were normalised to the [^3^H]-glycine released from un-treated wells (Basal) and fitted to a four-parameter equation.

nBoNT/F1, rBoNT/F1, mrBoNT/F1^R1111K^ and mrBoNT/F7-1 all produced concentration-dependent decreases in the release of [^3^H]-glycine from eSCN following BoNT intoxication (Figure 3).

In this assay, rBoNT/F1 showed a slight albeit significantly lower potency than nBoNT/F1 (pIC_50_ 9.32 ± 0.06 and 9.62 ± 0.08 respectively). mrBoNT/F1^R11111K^ (9.71 ± 0.06) had a similar activity to nBoNT/F1 and mrBoNT/F7-1 had the highest potency (10.58 + 0.03; Table 4). 

The same residual neurotransmitter release was observed for the four BoNTs. (14.80 ± 2.99 *n* = 4, 13.85 ± 1.49 *n* = 4; 13.85 ± 2.05 *n* = 3 and 13.00 ± 1.49 *n* = 3 for nBoNT/F1, rBoNT/F1, mrBoNT/F1^R11111K^ or mrBoNT/F7-1, respectively, mean ± SEM n experiments run in triplicate). For comparison BoNT/A has a pIC_50_ of 12.42 ± 0.06 [13].

### 2.4. Inhibition of Mouse Hemi-Diaphragm Contraction

**Figure 4:**
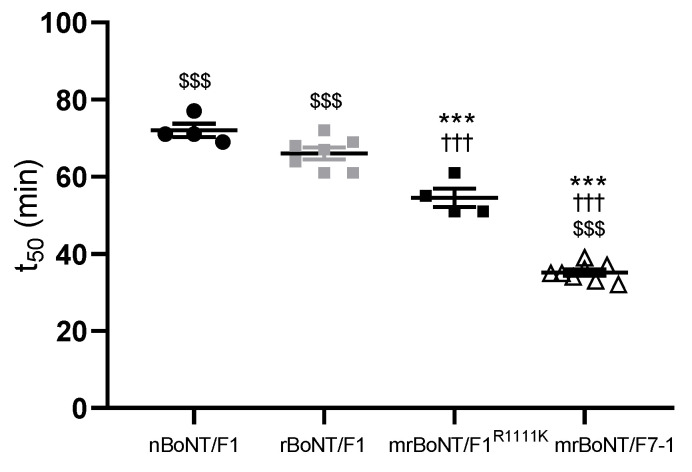
Inhibition of striatal muscle activity in mouse phrenic nerve hemidiaphragm preparation time to inhibit 50% of the initial force contraction (t_50_) following treatment with 100 pM of nBoNT/F1, rBoNT/F1, mrBoNT/F1^R1111K^ or mrBoNT/F7-1. *** *p* < 0.001 vs. nBoNT/F1, ^†††^ *p* < 0.001 vs. rBoNT/F1, ^$$$^ *p* < 0.001 vs. mrBoNT/F1^R1111K^.

nBoNT/F1, rBoNT/F1, mrBoNT/F1^R1111K^ and mrBoNT/F7-1 produced time-dependent decreases in the muscle contractility of mouse PNHD. At 100 pM, nBoNT/F1 and rBoNT/F1 had the highest t_50_ of 72 ± 2 and 66 ± 2 min, respectively. Interestingly, mrBoNT/F1^R1111K^ displayed a significantly lower t_50_ of 55 ± 2 min in comparison to either natural or recombinant BoNT/F1. mrBoNT/F7-1 was the most potent in this assay with a t_50_ of 35 ± 1 min. (Figure 4 and Table 5). Previously measured, the t_50_ of BoNT/A was 27.20 ± 0.66 min at 100 pM [13].

### 2.5. Inhibition of Detrusor Smooth Muscle Contraction

**Figure 5:**
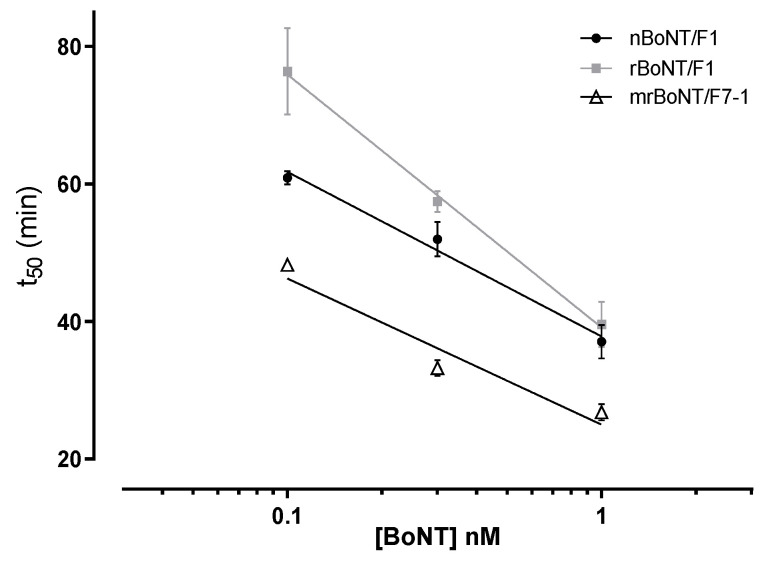
Inhibition of detrusor smooth muscle contraction in the mouse bladder strip assay (A) shows concentration-dependent time to inhibit 50% of the initial force contraction (t_50_) following treatment with nBoNT/F1, rBoNT/F1, or mrBoNT/F7-1.

Previously, the mouse detrusor assay had shown a different sensitivity between BoNT/A and BoNT/B, in contrast to their similar activities in the mouse phrenic nerve hemidiaphragm preparation [24]. Furthermore, nBoNT/F1 was shown to be even more potent than serotype A and B in this assay [25]. We assessed the potency of these molecules and showed that they blocked EFS-induced detrusor contraction in a dose-dependent manner (Figure 5). 

They were all able to inhibit contractions in the tested concentration range, allowing logarithmic functions fitting with excellent R^2^ values (nBoNT/F1; 0.99; rBoNT/F1, 1 and mrBoNT/F7-1, 0.94). Relative to nBoNT/F1, rBoNT/F1 displayed a slightly lower potency while mrBoNT/F7-1 showed a greater potency in this assay (Table 6). 

Previously, the estimated concentration to generate a t_50_ of 70 min for BoNT/A was 2.438 nM [25]. The data here show both recombinant sub-serotypes were also more potent than BoNT/A.

### 2.6. Inhibition of the Digit Abduction in Mouse

**Figure 6:**
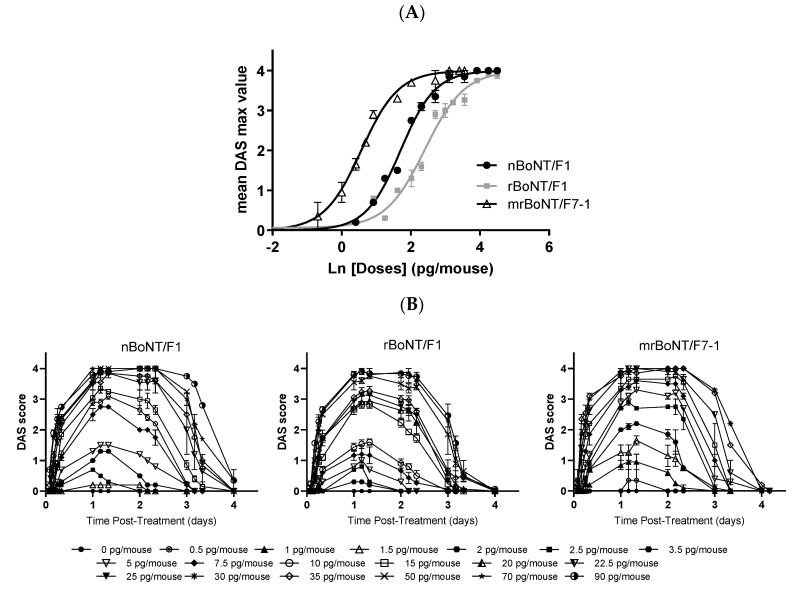
DAS assay in mice (**A**) Dose–response curve after intramuscular injection of nBoNT/F1, rBoNT/F1 and mrBoNT/F7-1. Fitting with a 4-parameter logistic equation with the bottom parameter constrained to zero and the top parameter constrained to 4. Each point represents the mean ± SEM of 2/3 experiments with *n* = 6 animals per dose. (**B**) DAS value time-course after injection into the gastrocnemius of mice, each point represents means of 6 values ± SEM of *n* = 2–3 experiments.

In mice, acute, i.m. administration of either nBoNT/F1 (1.5–90.0 pg/mouse), rBoNT/F1 (2.5–90.0 pg/mouse) or mrBoNT/F7-1 (0.5–35.0 pg/mouse) caused rapid and dose-related increases in DAS values (Figure 6A). 

The mean ED_50_ values were 5.5 [5.11–6.02], 11.2 [10.00–12.64] and 1.8 [1.62–1.97] pg/animal, for nBoNT/F1, rBoNT/F1 or mrBoNT/F7-1, respectively (Table 7).

Both rBoNT/F1 and mrBoNT/F7-1 ED_50_ values were significantly different from nBoNT/F1 and mrBoNTF7-1 ED_50_ was also significantly lower than the one of rBoNT/F1. Mice treated with these BoNTs showed rapid inhibition of the digit abduction, starting at 2 h after injection for the doses of 35.0, 50.0 and 15.0 pg/mouse for nBoNT/F1, rBoNT/F1 and mrBoNT/F7-1, respectively. DAS responses were maximal (DAS 4) between D1 and D2 and rapidly started to decrease and animals had recovered within D4. DAS 4 dose (50.0, 70.0 and 22.5 pg/mouse for nBoNT/F1, rBoNT/F1 and mrBoNT/F7-1, respectively) showed similar time-course profile (Figure 6B). For comparison, BoNT/A has an ED_50_ value of 2.3 pg/mouse with a maximal DAS 4 score being reached in 3 days [13]. The recombinant and native BoNT/F are both shown to be less potent than BoNT/A. In contrast, mrBoNT/F7-1 has a higher measured potency in the same assay.

**Figure 7:**
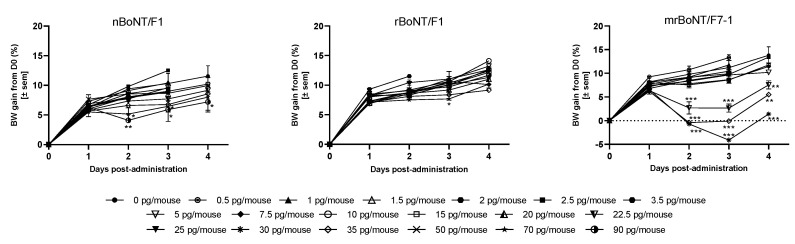
Bodyweight (BW) change in the following days after BoNTs administration. Mean ± SEM of 2/3 experiments with *n* = 6 animals per dose, * *p* < 0.05, ** *p* < 0.005 and *** *p* < 0.001in comparison to the vehicle-treated animals. One-way ANOVA followed by Dunnett’s.

All the BoNTs affected body weight (BW) gain during the study and in a dose-related manner. From the dose of 0.5 to 15.0 pg/mouse, none of the BoNTs affected the BW gain in comparison to vehicle-treated animals. For nBoNT/F1, BW gain remained comparable until 50.0 pg/mouse, then, starting at D2 after injection, animals treated with 70.0 to 90.0 pg/mouse of nBoNT/F1 gained significantly less weight than vehicle-treated animals and this persisted until the end of the experiment (D4). In contrast, the 70 pg/mouse rBoNT/F1 treated animals transiently gained less weight than the vehicle-treated animals while no other dose treatments affected the BW gain. Finally, we observed a more pronounced change in the BW gain following treatment with mrBoNT/F7-1. From 22.5 pg/mouse, mrBoNT/F7-1 treated animals significantly lost weight between D2 and D3 and started to recover at D4 (Figure 7). 

In one of the two experiments with mrBoNT/F7-1, animals treated with 30.0 and 35.0 pg/mouse displayed a severe body weight loss, associated with clinical signs such as ruffled fur, abdominal ptosis and palpebral ptosis. They were humanely euthanised before the experiment end and data were excluded from the analysis.

In order to compare BoNTs safety margins, we calculated their Therapeutic Index (Table 7). The one of rBoNT/F1 appeared a half lower than the one of mrBoNT/F7-1, which was comparable to the one of nBoNT/F1.

## 3. Discussion

Although rarely responsible for Botulism cases in humans, the serotype F botulinum neurotoxin family encompasses a high sequence diversity with strains isolated from different sources and geographical sites. For instance, recently, two foodborne cases were reported: the first in an infant in Africa from a rare bivalent Clostridium botulinum strain, producing subtype B3 and F8 toxins [26], and the second in an adult caused by type B and F [27]. These differences translate especially in differential substrate recognition and cleavage mechanism [2,19,20].

To study BoNT/F7 in more detail, we used an *E.coli* system which is now a well-established method that offers control over expression and purification processes. The functional characterisation of the protein requires the protein’s integrity to be maintained at each step of the process. One critical step is the post-translational proteolytic cleavage which produces the fully active di-chain form of the BoNT. Based on an approach reported in a previous study [23], we introduced the BoNT/F1 activation loop into the BoNT/F7 sequence and successfully produced mrBoNT/F7-1. Most of the work on BoNT/F7 reported to date has been focused on the LC and differences in enzymatic activity. The HC structure also supports potential key differences but much less work has so far been carried out to investigate these aspects. Benson et al. identified two arginine residues as key to coordinating a critical sugar residue of GD1a dusring binding [22]. Sequence alignment shows that the equivalent residue to one of these two arginines in BoNT/F1 is substituted with lysine in the BoNT/F7 sequence. In order to assess whether this difference changed the activity in functional assays the mutant mrBoNT/F1^R1111K^ was created. 

The specific activity of mrBoNT/F7-1 LC in the HPLC VAMP-2 cleavage assay was significantly higher than any other BoNT/F tested. This is consistent with previous findings. Indeed, although F7 substrate recognition differs from F1, requiring more extensive interactions with the VAMP-2 exosite [19,20], Sikorra et al. showed that the K_cat_ was 1.5-fold higher than the one of F1 [2]. mrBoNT/F1^R1111K^ had comparable activity to the one of nBoNT/F1 or rBoNT/F1, showing that the mutated residue in the ganglioside binding domain had, as expected, no impact on the catalytic activity.

The enhanced activity of mrBoNT/F7-1 was further confirmed in the glycine release assay. The potency was significantly higher than any other BoNTs tested. Interestingly, mrBoNT/F1^R1111K^ potency was slightly higher than that of rBoNT/F1, suggesting that the mutated residues could have a positive effect on its activity in this assay. The difference was even more pronounced in the mouse phrenic nerve hemidiaphragm assay, in which mrBoNT/F1^R1111K^ was significantly more potent than natural or recombinant BoNT/F1 but less than mrBoNT/F7-1. 

A homology model of the BoNT/F7 binding domain was constructed and compared to that of the previously reported BoNT/F1-GD1a complex (Figure S3). Here the lysine was not shown to specifically interact with the sugar residue. In fact, the surrounding residues would suggest an altogether more flexible region, so the modelling did not provide an explanation for the experimental data. It could be postulated that the mutation arginine to lysine is not significant, but the flexibility of the region is important. A lysine adds less stability to the protein structure, reinforcing the flexibility. It could improve the binding of the loop to the ganglioside or even allow binding to a greater range of gangliosides. This could then translate into a greater amount of LC being internalised and translocated, hence an increased potency in these assays. Further investigation with the corresponding mutant lysine to arginine mutant in the BoNT/F7 binding domain would be of interest.

To investigate potency at the neuromuscular junction (NMJ) in an in vivo assay, we examined potency, duration and therapeutic index of mrBoNT/F1 in the mouse DAS assay.

Similar to results from the ex vivo hemidiaphragm assay, mrBoNT/F1 displayed a greater potency at the NMJ level as it induced full paresis (DAS 4) at lower BoNT doses in comparison to nBoNT/F1 or BoNT/F1. Interestingly, this increased potency did not affect the time course as the digit abduction inhibition occurred within 2 h of injection and was resolved by day 4 after injection. This suggests that the enhanced catalytic activity of mrBoNT/F7-1 does not alter a prominent characteristic of the serotype F, which is described as medium-lasting neurotoxin [7]. This is in contrast with a study of different subtypes of serotype A, in which it was shown that the A3 subtype had a shorter duration and less potent LC protease activity compared to A1 [28]. In this study, we observed that although more potent at the level of proteolytic activity, mrBoNT/F7-1 had the same duration in vivo as nBoNT/F1. Within a serotype, different subtypes can have distinct potencies and durations but it is not yet possible to infer one from the other. 

Another interesting characteristic of mrBoNT/F7-1 is its superior ability to inhibit detrusor muscle contraction. Indeed, it was 5.6-fold more potent than the native BoNT/F1 in the mouse bladder strip assay. In contrast with the mouse phrenic nerve hemidiaphragm assay, it was shown that BoNT/F was more effective than serotype A or B in this assay. Additionally, BoNT/F was also more potent in the preparation of human detrusor strip assays [25]. These data suggest that mrBoNT/7-1 could be of interest in the development of a treatment involving autonomic disorders.

Using the therapeutic index as an indicator for safety, we showed that whilst mrBoNT/F7-1 has an enhanced potency, its therapeutic index remains similar to that of nBoNT/F1. It suggests that the enhanced potency did not increase spread compared to F1. One physicochemical property involved in tissue retention is the overall predicted isoelectric point (p*I*), which is 7.44 and 5.59 for BoNT/F7 and BoNT/F1, respectively. The difference in the isoelectric point is substantial and it is well documented that an increase in positive charge at physiological pH can help with tissue retention [29]. The high p*I* of mrBoNT/F7 may then induce increased retention of the molecule within the muscle tissue and therefore limit the systemic spreading. Further studies would be required to get a better understanding. Nonetheless, it was reported several times in preclinical studies that the serotype F had the highest safety margins in comparison to other serotypes [12,30] which makes this serotype of great interest for the development of new therapeutics.

## 4. Materials and Methods

### 4.1. Materials

Oligonucleotides were synthesised by Eurofins Genomics. The expression plasmid pJ401 was synthesised at DNA 2.0 and the expression plasmid pK8 (pET26b derived) was synthesised at Entelechon. All other reagents were from Sigma-Aldrich unless stated otherwise.

### 4.2. Molecular Cloning

The codon-optimised, synthetic genes encoding the BoNT/F1 protein (uniparc: UPI00000B66D1) and the BoNT/F7 protein (uniparc: UPI0001DE3DAC) were synthesised as two parts (DNA 2.0) in each case and subcloned into either pK8 vector (BoNT/F1) or pJ401 vector (BoNT/F7). The constructs mrBoNT/F7-1, mrBoNT/F7-1(0) and rBoNT/F1^R1111K^ were created by SDM (site-directed mutagenesis) using the quick-change method (Merck Millipore). The SDM primer sequences were: F7_1_For CCAAGAAGGGTACGAAAGCACCGCCTCGCCTGTGCATTAAAGTCAAC, F7_1_RevGTTGACTTTAATGCACAGGAGAGGCGGTGCTTTCGTACCCTTCTTGG, F72_For CTTCGTGGGCCTGTGCAAAGCGTTATTCCGCGTAAGGGTACGAAGCAC, F7_2_Rev GTGCTTTCGTACCCTTACGCGGA ATAACGCTTTTGCACAGGCCCACGAAG, F7(0)_For CGATCTCCCTGGCCCATCACTGTCTACGTCTTGCACGG, F7(0)_Rev AGAGGGACCGGGTAGTTGACTAGATGCAGAACGTGCCAGACATGCCA, 1RK1111F CTATCTGCTGAATCTGTTGAAGACCGACAAG and 1RK1111R GTTTTGGGTAATGCTCTTGTCGGTCTTCAACAG. Sanger sequencing analysis was performed to confirm each nucleotide sequence generated.

### 4.3. Protein Expression and Purification

The constructs were expressed as previously detailed [23]. Briefly, an expression strain of *E. coli* was transformed with a plasmid containing a rBoNT/F construct. Single colonies were picked to inoculate a shake flask culture. The cultures were allowed to reach an OD600 of 1.0 before reducing the temperature 16 °C and chemically inducing with IPTG. Cultures were incubated for a further 20 h and harvested by centrifuge. The Biomass was collected and stored at −80 °C until required. The protein was then extracted and further purified as follows: Cell pellets were lysed by sonication or homogenised in a high-pressure cell disruptor in lysis buffer (50 mM Tris (pH 8), 200 mM NaCl), supplemented with Bezonase^®^ Nuclease (1:17667) (Sigma-Aldrich) the lysate was incubated for 20 min before adjusting the buffer to 50 mM Tris (pH 8), 100 mM NaCl, 1.3 M (NH_4_)_2_SO_4_ by the addition of an equal volume of 50 mM Tris (pH 8), 1.6 M (NH_4_)_2_SO_4_. The lysate was then clarified by centrifugation. Clarified lysate was loaded onto a Butyl HP column (GE Healthcare, Little Charpentine, UK) on an ÄKTA Pure (GE Healthcare). The column was washed with 50 mM Tris (pH 8), 1.35 M (NH_4_)_2_SO_4_. The remaining bound protein was eluted over a linear gradient down to 50 mM Tris (pH 8). Eluted BoNT/F was buffer exchanged on a Hiprep 26/10 desalting column into 50 mM Tris pH 8.5 and loaded onto a Q HP HiTrap column (GE Healthcare). The column was washed with 50 mM Tris (pH 8.5). The remaining bound protein was eluted over a linear gradient to 50 mM Tris (pH 8.5), 300 mM NaCl. Eluted BoNT/F was buffer exchanged into activation buffer (Gibco^®^ PBS (pH 7.2)). The single-chain BoNT/F was treated with 0.2 µg/mL Endoproteinase Lys-C (Sigma-Aldrich) at 4 °C for 20 h. The BoNT/F buffer salt concentration was adjusted to 3M NaCl with 1.6 volumes of 5 M NaCl and loaded onto Phenyl HP HiTrap column (GE Healthcare). The column was washed with 10 mM Tris pH 8, 3 M NaCl and the remaining bound protein was eluted over a linear gradient down to 10 mM Tris pH 8. The eluted BoNT/F was buffer exchanged into Gibco^®^ PBS (pH 7.2). The protein was stored at stock concentration (final formulation: Gibco^®^ PBS (pH 7.2)) or 0.1 mg/mL (final formulation: Gibco^®^ PBS (pH 7.2) + 1 mg/mL BSA) at −80 °C. All Neurotoxin manipulations were performed in ACDP level 2 laboratories within microbiological safety cabinets.

### 4.4. SDS-PAGE

In-process samples and final product samples were analysed by SDS-PAGE. Consumables and methods used are as previously stated [23]. Image analysis was performed using Syngene software genesnap and genetools.

### 4.5. Cell-Free VAMP-2 Proteolysis AssayE

mrBoNT/F7-1, rBoNT/F1, mrBoNT/F1^R1111K^ and nBoNT/F1 were diluted to 1 nM in assay buffer (50 mM HEPES pH 7.2, 20 µM ZnCl_2_, 0.2 mg/mL BSA, 20 mM DTT). The soluble region of human VAMP-2 (amino acids 2-94) was expressed and purified as previously reported [23]. The VAMP-2(2-94)-GFP was then diluted to 50.5 µM in assay buffer. Each diluted BoNT sample was mixed with an equal volume of VAMP-2(2-94)-GFP and incubated for 30 min at 37 °C. Reactions were terminated by the addition of 0.25 volumes of 0.3 M EDTA. Reaction mixtures were then further diluted with 125 µL of sample buffer (0.1% TFA in water). Samples were then run on a C3 reverse-phase analytical column, Agilent using an Agilent 1100 series HPLC. The samples were separated on a gradient using sample buffer and elution buffer (0.1% TFA in Acetonitrile). Substrate and product peaks were integrated and compared to determine specific activity. 

### 4.6. Assessment of Glycine Release from Rat Embryonic Spinal Cord Neurons Treated with BoNTs

Rat embryonic spinal cord neurons (eSCN) were prepared from spinal cords dissected from embryonic day 15 (E15) Sprague Dawley rats as described previously [13]. Glycine release was assessed in eSCN cultured in 96 well plates until DIV 20–23. eSCN were treated with a concentration range of BoNT for 24 h at 37 °C. Following removal of neurotoxin, cells were briefly washed 3 times in HEPES-buffered salt solution (136 mM NaCl, 3 mM KCl, 2 mM CaCl_2_, 1 mM MgCl_2_, 10 mM HEPES, 10 mM glucose, pH 7.2). Cells were loaded with 2 µCi/mL [^3^H]-glycine (Perkin Elmer, Beaconsfield, UK) in HBS for 60 min at 35 °C. Following removal of [^3^H]-glycine, cells were briefly washed 3 times with HBS. Basal and stimulated [^3^H]-glycine releases were measured by incubation at 35 °C for 5 min with 50 µL/well of HBS solutions containing low (3 mM KCl) or high (60 mM KCl) potassium. To determine retained [^3^H]-glycine in the cells, cells were lysed by adding 50 µL/well RIPA buffer (Sigma). Superfusates and cell lysates were transferred into 96-well Isoplates (Perkin Elmer) and 200 µL/well OptiPhase Supermix scintillation fluid was added. Radioactivity was quantified using a MicroBeta2 plate reader (Perkin Elmer). Data were fitted to a 4-parameter logistic equation, and pIC_50_ and IC_50_ were calculated.

### 4.7. Mouse Phrenic Nerve Hemidiaphragam Assay

Isolated, mouse left hemi-diaphragms were obtained from adult male, CD-1 mice purchased from Charles River (Margate, UK). With the phrenic nerve attached, they were incubated in a tissue bath (Linton Instrumentation, Roydon, UK) containing gassed Krebs-Henseleit buffer (118 mM NaCl, 1.2 mM MgSO_4_, 11 mM glucose, 4.7 mM KCl, 1.2 mM KH_2_PO_4_, 2.5 mM CaCl_2_, 25 mM NaHCO_3_, pH 7.5). The phrenic nerve was continuously electro-stimulated (10 V, 20 µs, 1 Hz) and the resultant isometric contractions of the diaphragm muscle were recorded. Following a period of stability, the tissue was incubated with 3 µM tubocurarine hydrochloride (Sigma), a competitive antagonist at nicotinic acetylcholine receptors on post-synaptic muscle cells, until >95% paralysis of the muscle was observed. This provided confidence that the diaphragm muscle contraction was due to acetylcholine release from pre-synaptic neurons rather than direct electrical stimulation of the muscle through the buffer. Tubocurarine was washed out, and following a further period of stability, the control contractile tension of the muscle was recorded before the assay buffer was exchanged for buffer containing BoNT. Muscle activity was recorded until no further muscle contraction was detected. The potency of BoNTs was estimated as the time to reduce the amplitude of diaphragm contraction to 50% (t_50_) of control following BoNT addition. Following complete paralysis, the muscle was directly stimulated by increasing the strength of the applied electro-stimulation in order to establish continued physiological viability of the preparation.

### 4.8. Mouse Detrusor Strip Assay

The detrusor strip assay was performed as previously described [24]. Briefly, mouse bladders from female C57 Bl6/N mice from Janvier Labs (Saint Berthevin, France) were dissected up to the detrusor muscle and 2 strips were cut out of it. They were tensed in tissue baths and subjected to electrical field stimulation with trains of 20 pulses (10 Hz, 20 µsec) to generate neurogenic contractions. Prior and after BoNTs testing, carabachol was used to ensure postsynaptic response and experiments with post-stimulation carbachol responses <80% of the pre-stimulation response were rejected. After washing, BoNTs (0.1; 0.3 and 1 nMn = 3–4 strips per concentration) were added to the baths (one strip per concentration) and contraction amplitude was measured until 90% of the signal was abolished. For each concentration, t_50_ (time to reach half paralysis) was determined and plotted against the concentration. Using linear regression, the theoretical t_50_ of 70 min was calculated [31] and relative potency in reference to nBoNT/F1 was expressed.

### 4.9. Mouse Digit Abduction Score (DAS) Assay 

Adult male CD-1 mice (24–30 g) from Charles River (Saint-Germain-Nuelles, France) were anaesthetised with 4% isoflurane/oxygen in an induction chamber before administration of an i.m. injection of either BoNT or vehicle (gelatin phosphate buffer, GPB). The injections were performed in a 20 µL volume into the gastrocnemius-soleus muscle complex of the right hind limb using a 30-gauge needle attached to a 100 µL syringe (SGE Analytical Science, Interchim; Montluçon, France). nBoNT/F1 (Metabiologics), rBoNT/F1 and rBoNT/F7-1 were aliquoted at 0.1 mg/mL in phosphate-buffered saline (KH_2_PO_4_ 1 mM, Na_2_HPO_4_ 3 mM, NaCl 155 mM, pH 7.4), stored at −80 °C and a new aliquot was used for each experiment. BoNTs were diluted to yield final concentration in GPB, containing 50 mM Na_2_HPO_4_ (Emsure^®^, Merck, Darmstadt, Germany), 0.2 % (*v*/*v*) gelatin (Prionex^®^, Sigma) and sterile water (Ecotainer^®^, Aqua B. Braun, Melsungen, Germany), the pH was adjusted to 6.5 with orthophosphoric acid (85%, Merck). Effects of each BoNT serotype in mice were assessed in independent experiments. Specifically, mice (*n* = 6/dose) were assessed for normal DAS response before being treated with either BoNTs. nBoNT/F1 was injected at 1.5, 2.5, 3.5, 5.0, 7.5, 10.0, 15.0, 22.5, 35.0, 50.0, 70.0 and 90.0 pg/mouse, rBoNT/F1 at 2.5, 3.5, 5.0, 7.5, 10.0, 15.0, 20.0, 25.0, 35.0, 50.0, 70.0 and 90.0 whereas rBoNT/F7-1 was injected at 0.5, 1.0, 1.5, 2.0, 2.5, 5.0, 7.5, 15.0, 2.5, 30.0 and 35.0 pg/mouse (*n* = 6 animals/dose/experiment). Each experiment also included a control group of animals injected only with vehicle, GPB (*n* = 6). At the end of the experiment, or earlier if deemed necessary, animals were euthanised by CO_2_ asphyxiation.

The mouse DAS test was performed as previously described [32]. Mice were suspended briefly by the tail to elicit a typical startle response characterised by the hind limb extension and abduction of digits. The mouse DAS was assessed using a five-point scale from DAS 0 = normal to DAS 4 = maximal reduction in digit abduction and leg extension. Mice were scored for DAS response at baseline (day 0) and daily on days 1–4 by an observer blind to the treatment. DAS 4 dose was defined as the first experimental dose inducing the mean DAS value of 4 for that dose group. For analysis, each dose (pg/animal) was converted to its natural logarithm and the highest average DAS score (mean DAS max) per dose group was plotted. The data were fitted to a 4-parameter logistic equation curve fit, with the lower asymptote constrained to zero and the upper asymptote constrained to 4. The obtained dose value corresponding to half-maximal DAS (DAS 2) from the equation was converted to ED_50_ by inverting the calculated value in the natural logarithm scale to its linear value. Bodyweight (BW) changes and clinical signs were recorded daily throughout the study. Data are expressed as percentage of BW changes from the day of injection (D0).

### 4.10. Data and Statistical Analysis

The concentration–response and dose–response curves analyses were performed using GraphPad Prism version 7.04 (GraphPad Software Inc., La Jolla, CA, USA).

Statistical differences were determined by one-way ANOVA, followed by appropriate post-hoc test using GraphPad Prism. Significance was determined at the level *p* < 0.05.

## 5. Patents

US2018/0073089 A1 granted 12-05-2020.

## Figures and Tables

**Table 1:** N-terminal sequencing of mrBoNT/F7-1 HC subunit excised from an SDS-PAGE gel where “?” represents an inconclusive signal and “-” indicates no signal generated.

	N-Terminal Residues of mrBoNT/F7-1						
	1	2	3	4	5	6	7
HC (weak signal) Observed	A?	P	P	-	L?	-	L?
predicted	A	P	P	R	L	C	I

**Table 2:** LC-MS intact mass analysis of mrBoNT/F7-1 (0) for both full-length protein and reduced sub-units. Mass is expressed in daltons.

		LC-MS Intact Mass Data of mrBoNT/F7-1 (0)			
Full-Length		LC		HC	
Predicted	Observed	Predicted	Observed	Predicted	Observed
145,677.4	145,707	48,730.1	48,722	96,965.3	96,985

**Table 3:** Specific activity and replicate for each compound.

Protein	N (Replicates)	Average Specific Activity	Average Standard Deviation	Average % Potency
nBoNT/F1	6	35,661.4	6386.6	100.0
rBoNT/F1	3	24,325.9	598.0	75.2
mrBoNT/F1^R1111K^	3	28,455.1	1796.3	94.8
mrBoNT/F7-1	3	68,291.0	9836.8	165.5 ***^,†††,$$$^

*** *p* < 0.001 vs. nBoNT/F1, ^†††^ *p* < 0.001 vs. rBoNT/F1, ^$$$^ *p* < 0.001 vs. mrBoNT/F1^R1111K^. One-way ANOVA followed by Dunnett’s post-hoc test.

**Table 4:** Concentration required for 50% of maximal inhibition (pIC_50_) for each protein and number (*n*) of independent experiments.

Protein	pIC_50_
nBoNT/F1	9.62 ± 0.08 (*n* = 4)
rBoNT/F1	9.32 ± 0.06 (*n* = 4) *
mrBoNT/F1^R1111K^	9.71 ± 0.06 (*n* = 3) ^††^
mrBoNT/F7-1	10.58 ± 0.03 (*n* = 3) ***^,†††,$$$^

* *p* < 0.05, *** *p* < 0.001 vs. nBoNT/F1, ^††^ *p* < 0.01 and ^†††^ *p* < 0.001 vs. rBoNT/F1, ^$$$^ *p* < 0.001 vs. mrBoNT/F1^R1111K^. Data are means ± SEM. One-way ANOVA followed by Tukey post-hoc test.

**Table 5:** t_50_ (time to 50% inhibition) values from hemi-diaphragm contraction assay.

Protein	t_50_ (Min)
nBoNT/F1	72 ± 2 ^$$$^ (*n* = 4)
rBoNT/F1	66 ± 2 ^$$$^ (*n* = 7)
mrBoNT/F1^R1111K^	55 ± 2 ***^,†††^ (*n* = 4)
mrBoNT/F7-1	35 ± 1 ***^,†††,$$$^ (*n* = 8)

*** *p* < 0.001 vs. nBoNT/F1, ^†††^ *p* < 0.001 vs. rBoNT/F1, ^$$$^ *p* < 0.001 vs. mrBoNT/F1^R1111K^. Data are means ± SEM and number (*n*) of independent experiments. One-way ANOVA followed by Tukey post-hoc test.

**Table 6:** Estimated concentration to reach theoretical t_50_ values of 70 min using logarithmic function curve and the relative potency normalised from nBoNT/F1.

Protein	Estimated [BoNT] Resulting in t_50_ of 70 Mins (nM)	Potency Relative to nBoNT/F1
nBoNT/F1	0.045	1.0
rBoNT/F1	0.145	0.3
mrBoNT/F7-1	0.008	5.6

**Table 7:** ED_50_, DAS4 dose, effect on the BW expressed as the 1st dose with a significant effect on the BW following acute, i.m. administration of nBoNT/F1, rBoNTF1 or mrBoNT/F7-1 in CD-1 mice.

Protein	ED_50_ (pg/Animal)	DAS4 Dose (pg/Animal)	1st Dose with Significant Effect on the BW (In Comparison to Vehicle Treated Group)	TI (Dose with Effect on the BW/ED_50_)
nBoNT/F1	5.5 [5.1–6.02]	50	^#^ 70	12.7
rBoNT/F1	11.2 *** [9.95–12.71]	70	^#^ 70 (transitory effect)	6.25
rBoNT/F7-1	1.8 ***^,†††^ [1.62–1.97]	22.5	^###^ 22.5	12.5

*** *p* < 0.001 vs. nBoNT/F1, ^†††^ *p* < 0.001 vs. rBoNT/F1; ^#^ *p* < 0.05 and ^###^ *p* < 0.001 against the vehicle-treated group. The Therapeutic Index (TI) of a molecule was determined by dividing the 1st dose with effect on the BW gain with the ED_50_.

## Supplementary Materials

The following are available online at https://www.mdpi.com/article/10.3390/toxins13120834/s1, Figure S1: Sequence alignment of BoNT/F1 and BoNT/F7; Figure S2: SDS-PAGE analysis shows test activation of rBoNT/F7 (0) with various proteases; Figure S3: Shows a homology model of the BoNT/F7 GBM aligned with the crystal structure of the BoNT/F1-GD1a complex.
