# Supplementary material for: New Modified Recombinant Botulinum Neurotoxin Type F with Enhanced Potency
Source: Toxins (Basel). 2021 Nov 24;13(12):834. doi: 10.3390/toxins13120834 (PMC8705745; doi:10.3390/toxins13120834)
Supplement: Supplementary file 1 [file toxins-13-00834-s001.zip › toxins-1457720-supplementary.pdf]

Supplementary Materials: New Modified Recombinant Botulinum Neurotoxin Type F with Enhanced Potency

David Burgin, Cindy Périer, Gavin Hackett, Mark Elliot, Daniel Kwan, Fraser Hornby, Imran Mir, Jacquie Maignel, Sai Man Liu and Matthew Beard

```
BoNT/F1. MPVVINSFNYNDPVNDDTILYMQIPYEEKSKKYYKAFEIMRNVWIIPERNTIGTDPSDFD 60
BoNT/F7. MPVNINNFNYNDPINNTTILYMKMPYYEDSNKYYKAFEIMDNVWIIPERNIIGKKPSDFY 60
         *** **.******:*: *****::** *.*:********* ********* **..****
BoNT/F1. PPASLENGSSAYYDPNYLTTDAEKDRYLKTTIKLFKRINSNPAGEVLLQEISYAKPYLGN 120
BoNT/F7. PPISLDSGSSAYYDPNYLTTDAEKDRFLKTVIKLFNRINSNPAGQVLLEEIKNGKPYLGN 120
         ** **:.*******************:***.****:********:***:**. .******
BoNT/F1. EHTPINEFHPVTRTTSVNIKSSTNVKSSIILNLLVLGAGPDIFENSSYPVRKLMDSGGVY 180
BoNT/F7. DHTAVNEFCANNRSTSVEIKESKGTTDSMLLNLVILGPGPNILECSTFPVRIF-PNNIAY 179
         :** :***   .*:***:**.*.....*::***::** **:*:* *::*** :  .. .*
BoNT/F1. DPSNDGFGSINIVTFSPEYEYTFNDISGGYNSSTESFIADPAISLAHELIHALHGLYGAR 240
BoNT/F7. DPSEKGFGSIQLMSFSTEYEYAFND-------NTDLFIADPAISLAHELIHVLHGLYGAK 232
         ***:.*****::::** ****:***       .*: ***************.*******:
BoNT/F1. GVTYKETIKVKQAPLMIAEKPIRLEEFLTFGGQDLNIITSAMKEKIYNNLLANYEKIATR 300
BoNT/F7. GVTNKKVIEVDQGALMAAEKDIKIEEFITFGGQDLNIITNSTNQKIYDNLLSNYTAIASR 292
         *** *:.*:*.*. ** *** *::***:***********.: ::***:***:**  **:*
BoNT/F1. LSRVNSAPPEYDINEYKDYFQWKYGLDKNADGSYTVNENKFNEIYKKLYSFTEIDLANKF 360
BoNT/F7. LSQVNINNSALNTTYYKNFFQWKYGLDQDSNGNYTVNISKFNAIYKKLFSFTECDLAQKF 352
         **:**      : . **::********::::*.**** .*** *****:**** ***:**
BoNT/F1. KVKCRNTYFIKYGFLKVPNLLDDDIYTVSEGFNIGNLAVNNRGQNIKLNPKIIDSIPDKG 420
BoNT/F7. QVKNRSNYLFHFKPFRLLDLLDDNIYSISEGFNIGSLRVNNNGQNINLNSRIVGPIPDNG 412
         :** *..*::::  ::: :****:**::*******.* ***.****:** :*:. ***:*
BoNT/F1. LVEKIVKFCKSVIPRKGTKAPPRLCIRVNNRELFFVASESSYNENDINTPKEIDDTTNLN 480
BoNT/F7. LVERFVGLCKSIVSKKGTKN--SLCIKVNNRDLFFVASESSYNENGINSPKEIDDTTITN 470
         ***::* :***:: :****    ***:****:*************.**:********  *
BoNT/F1. NNYRNNLDEVILDYNSETIPQISNQTLNTLVQDDSYVPRYDSNGTSEIEEHNVVDLNVFF 540
BoNT/F7. NNYKKNLDEVILDYNSDAIPNLSSRLLNTTAQNDSYVPKYDSNGTSEIKEYTVDKLNVFF 530
         ***::***********::**::*.: *** .*:*****:*********:*:.* .*****
BoNT/F1. YLHAQKVPEGETNISLTSSIDTALSEESQVYTFFSSEFINTINKPVHAALFISWINQVIR 600
BoNT/F7. YLYAQKAPEGESAISLTSSVNTALLDASKVYTFFSSDFINTVNKPVQAALFISWIQQVIN 590
         **:***.****: ******::*** : *:*******:****:****:********:***.
BoNT/F1. DFTTEATQKSTFDKIADISLVVPYVGLALNIGNEVQKENFKEAFELLGAGILLEFVPELL 660
BoNT/F7. DFTTEATQKSTIDKIADISLVVPYVGLALNIGNEVQKGNFKEAIELLGAGILLEFVPELL 650
         ***********:************************* *****:****************
BoNT/F1. IPTILVFTIKSFIGSSENKNKIIKAINNSLMERETKWKEIYSWIVSNWLTRINTQFNKRK 720
BoNT/F7. IPTILVFTIKSFINSDDSKNKIIKAINNALRERELKWKEVYSWIVSNWLTRINTQFNKRK 710
         *************.*.:.**********:* *** ****:********************
BoNT/F1. EQMYQALQNQVDAIKTVIEYKYNNYTSDERNRLESEYNINNIREELNKKVSLAMENIERF 780
BoNT/F7. EQMYQALQNQVDGIKKIIEYKYNNYTLDEKNRLKAEYNIYSIKEELNKKVSLAMQNIDRF 770
         ************.**.:********* **:***::**** .*:***********:**:**
BoNT/F1. ITESSIFYLMKLINEAKVSKLREYDEGVKEYLLDYISEHRSILGN-SVQELNDLVTSTLN 839
BoNT/F7. LTESSISYLMKLINEAKINKLSEYDKRVNQYLLNYILENSSTLGTSSVQELNNLVSNTLN 830
         :***** **********:.** ***: *::***:** *: * **. ******:**:.***
BoNT/F1. NSIPFELSSYTNDKILILYFNKLYKKIKDNSILDMRYENNKFIDISGYGSNISINGDVYI 899
BoNT/F7. NSIPFELSEYTNDKILISYFNRFYKRIIDSSILNMKYENNRFIDSSGYGSNISINGDIYI 890
         ********.******** ***::**:* *.***:*:****:*** ************:**
BoNT/F1. YSTNRNQFGIYSSKPSEVNIAQNNDIIYNGRYQNFSISFWVRIPKYFNKVNLNNEYTIID 959
BoNT/F7. YSTNRNQFGIYSSRLSEVNITQNNTIIYNSRYQNFSVSFWVRIPKYNNLKNLNNEYTIIN 950
         *************: *****:*** ****.******:********* *  *********:
BoNT/F1. CIRNNNSGWKISLNYNKIIWTLQDTAGNNQKLVFNYTQMISISDYINKWIFVTITNNRLG 1019
BoNT/F7. CMRNNNSGWKISLNYNNIIWTLQDTTGNNQKLVFNYTQMIDISDYINKWTFVTITNNRLG 1010
         *:**************:********:**************.******** **********
BoNT/F1. NSRIYINGNLIDEKSISNLGDIHVSDNILFKIVGCNDTRYVGIRYFKVFDTELGKTEIET 1079
BoNT/F7. HSKLYINGNLTDQKSILNLGNIHVDDNILFKIVGCNDTRYVGIRYFKIFNMELDKTEIET 1070
         :*::****** *:*** ***:***.**********************:*: **.******
BoNT/F1. LYSDEPDPSILKDFWGNYLLYNKRYYLLNLLRTDKSITQNSNFLNINQQRGVYQKPNIFS 1139
BoNT/F7. LYHSEPDSTILKDFWGNYLLYNKKYYLLNLLKPNMSVTKNSDILNINRQRGIYSKTNIFS 1130
         ** .*** :**************:*******: : *:*:**::****:***:*.* ****
BoNT/F1. NTRLYTGVEVIIRKNGSTDISNTDNFVRKNDLAYINVVDRDVEYRLYADISIAKPEKIIK 1199
BoNT/F7. NARLYTGVEVIIRKVGSTDTSNTDNFVRKNDTVYINVVDGNSEYQLYADVSTSAVEKTIK 1190
         *:************ **** *********** .****** : **:****:* :  ** **
BoNT/F1. LIRTSNSNNSLGQIIVMDSIGNNCTMNFQNNNGGNIGLLGFHSNNLVASSWYYNNIRKNT 1259
BoNT/F7. LRRISNSNYNSNQMIIMDSIGDNCTMNFKTNNGNDIGLLGFHLNNLVASSWYYKNIRNNT 1250
         * * **** . .*:*:*****:******:.***.:******* **********:***:**
BoNT/F1. SSNGCFWSFISKEHGWQEN 1278
BoNT/F7. RNNGCFWSFISKEHGWQE- 1268
          .****************
```

Figure S1. Sequence alignment of BoNT/F1 and BoNT/F7. Key sites and motifs are highlighted in the F1 sequence as follows: Exosite 1 magenta; Exosite 2 blue; exosite 3 green; At or near active site red. The di-chain "activation loops" are highlighted in grey. The ganglioside binding domain is highlighted in dark yellow. Overall sequence identity 73.7%.

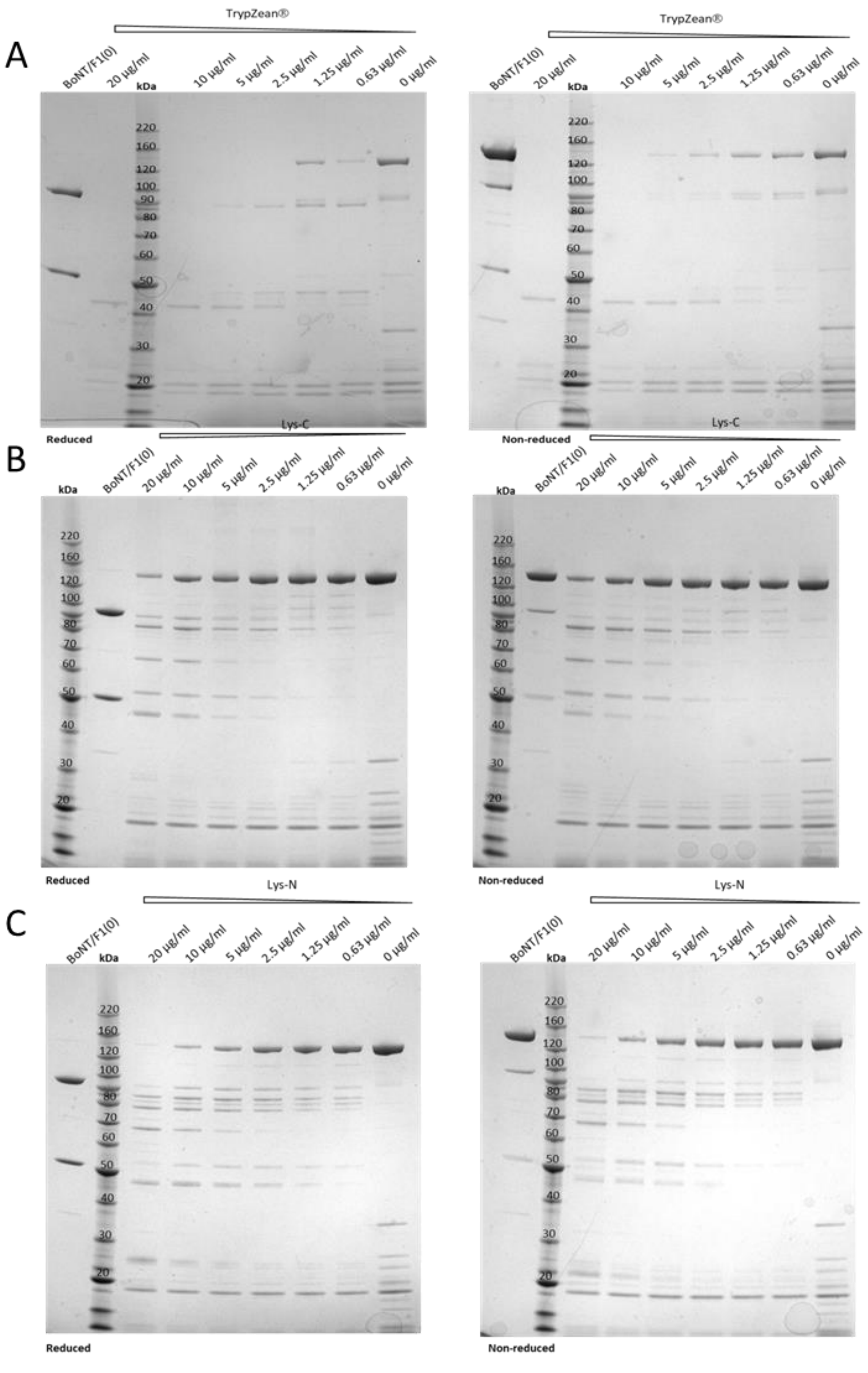

Figure S2. SDS-PAGE analysis shows test activation of rBoNT/F7 (0) with various proteases. In each case the digest was incubated for 2 h at 37 °C. On each gel Activated BoNT/F1 (0) was included as a positive control. (**A**) shows a protease digest with increasing concentrations of TrypZean® run as both reduced. (**B**) Shows a

protease digest with increasing concentrations of Lys-C run as both reduced and non-reduced samples. (**C**) Shows a protease digest run as both reduced and non-reduced samples.

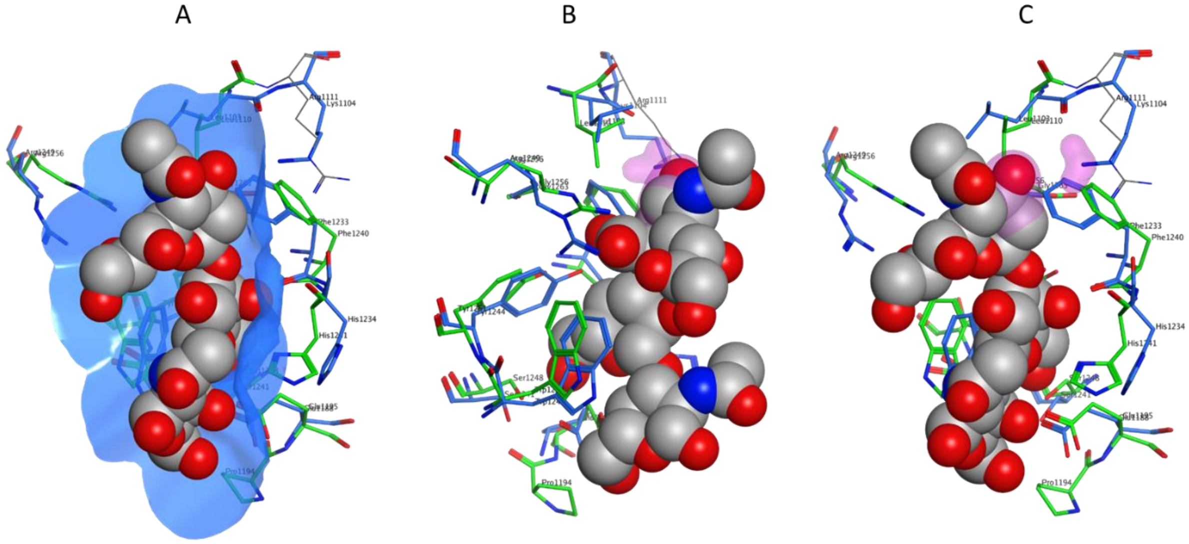

Figure S3. Shows a homology model of the BoNT/F7 GBM aligned with the crystal structure of the BoNT/F1-GD1a complex. (**A**) Shows the binding pocket. (**B**) Shows the left-hand view with potential interaction of Lys1104 (BoNT/F7) with GD1a. (**C**) Shows the right hand view with potential interaction of Lys1104 (BoNT/F7) with GD1a.
